# Supplementary material for: Mental health literacy in Arab states of the Gulf Cooperation Council: A systematic review
Source: PLoS One. 2021 Jan 7;16(1):e0245156. doi: 10.1371/journal.pone.0245156 (PMC7790272; doi:10.1371/journal.pone.0245156)
Supplement: S1 Appendix — (DOCX) [file pone.0245156.s001.docx]

# **SEARCH STRING**

| **Database** | **Search limitation** | **Concept** | **Search Term/strategy** | |
| --- | --- | --- | --- | --- |
|  |  |  | **Mesh OR Keywords** | |
| **PubMed**  **PsychInfo**  **Medline** | Up to June 2019  Adult  English  Search field: title, abstract and full text | #1 | “Health Literacy” OR “Health Knowledge, Attitudes, Practice” OR “Help-Seeking Behavior” OR “Attitude to health” OR “Social Stigma” | “Mental Health Literacy” OR “Mental health awareness” OR “Health knowledge” OR Knowledge OR “Mental Disorders Literacy” OR “Mood Disorders Literacy” OR “Depression Literacy” OR “Depressive Disorders Literacy” OR “Anxiety Literacy” OR “Bipolar Literacy” OR “Help seeking behaviour” OR “Help Seeking Behavior” OR “health seeking behaviour” OR “health seeking behavior” OR “seeking help” OR “help seeking” OR “Stigmatizing attitude” OR “Stigmatizing attitudes” OR stigma* attitude* OR “Attitude to health” OR percept* OR Believes |
|  |  | #2 | “Mental Disorders” OR “Anxiety Disorders” OR “[Depressive Disorder](https://www.ncbi.nlm.nih.gov/mesh/68003866)” OR “[[Mood](https://meshb.nlm.nih.gov/record/ui?ui=D019964) Disorder](https://www.ncbi.nlm.nih.gov/mesh/68003866)s” OR “Bipolar and Related Disorders” | “Disorder, Mental” OR “mental disorder*” OR “mental illness*” OR “[[Mood](https://meshb.nlm.nih.gov/record/ui?ui=D019964) Disorder](https://www.ncbi.nlm.nih.gov/mesh/68003866)*” OR “Depressive Disorder*” OR depression OR Depressi* OR “anxiety disorder*” OR anxiety OR schizophrenia OR “obsessive compulsive disorder*” OR “Bipolar disorders” OR dementia OR “Alzheimer disease” OR Alzheimer. |
|  |  | #3 | “Qatar” OR “Saudi Arabia” OR “Kuwait” OR “Oman” OR “United Arab Emirates” OR “Bahrain” | “GCC countries” OR “Gulf council countries” OR “Arab states” OR “Middle East” OR Qatar OR Bahrain OR Oman OR Kuwait OR Saudi Arabia OR KSA OR “United Arab Emirates” OR UAE OR Arab |

**#1 AND #2 AND #3**
