# Supplementary material for: Mental health literacy in Arab states of the Gulf Cooperation Council: A systematic review
Source: PLoS One. 2021 Jan 7;16(1):e0245156. doi: 10.1371/journal.pone.0245156 (PMC7790272; doi:10.1371/journal.pone.0245156)
Supplement: S1 Checklist — (DOC) [file pone.0245156.s002.doc]

| **Section/topic** | **#** | **Checklist item** | **Reported on page #** |
| --- | --- | --- | --- |
| **TITLE** | | |  |
| Title | 1 | Mental health literacy in Arab states of the Gulf Cooperation Council: A systematic review | 1 |
| **ABSTRACT** | | |  |
| Structured summary | 2 | **Aim**: Mental health literacy (MHL) has been relatively neglected, despite the increase of mental health illnesses worldwide, as well as within the Middle East region. A low level of MHL may hinder public acceptance of evidence-based mental health care. This systematic review aims to identify and appraise existing research, focusing on MHL among adults in the Gulf Cooperation Council (GCC) countries.  **Methods**: A systematic search of electronic databases (PubMed, PsychInfo, and Medline) was carried out from database inception to July 2019, in order to identify peer-reviewed journal articles that investigated MHL in the GCC countries. Studies were eligible for inclusion if they were: cross-sectional studies, reported in English, ‎targeted adults (aged 18 and above), conducted in any of the GCC countries, include at least one ‎outcome measure of the main components of MHL: knowledge of mental illnesses and their ‎treatment, stigmatizing attitudes towards mental illnesses, and seeking help for self and offering ‎help. ‎  **Results**: A total of 27 studies (16,391 participants) were included. The outcome across studies varied due to disparity in the tested populations. Findings show that limited MHL was observed among participants, even health care professionals. Results also show a high cumulative level of stigma and negative attitude towards mental health illness in the public. Negative beliefs and inappropriate practices are common, as well. The majority of studies yielded a moderate to high risk of bias.  **Conclusion**: This work indicates that research on MHL must be tackled through well-designed large-scale studies of the public. Campaigns to promote early identification and treatment of mental illness is also encouraged to improve overall level of MHL in the general population of the GCC region.  **Registration number**: PROSPERO 2018 CRD42018104492 | 2 |
| **INTRODUCTION** | | |  |
| Rationale | 3 | Since there are limited numbers of researches that reflect the situation of MHL in the Middle East region, the picture is still lacking for some clarity compared to the western world and African region. To our knowledge, there are no reports on systematic reviews that were conducted to assess the situation of MHL in the GCC countries. | 4 |
| Objectives | 4 | This systematic review aims to explore MHL in the GCC countries as well as to uncover similarities, differences, and ‎methodological issues among published studies.‎ | 4 |
| **METHODS** | | |  |
| Protocol and registration | 5 | Registration number for the study protocol is: PROSPERO 2018 CRD42018104492 | 2 |
| Eligibility criteria | 6 | Inclusion criteria were: (a) cross-sectional studies with no time restriction, (b) written in English, (c) age 18 and above and (d) conducted in any of the GCC countries (e) include at least one outcome measure of the main components of MHL which are: knowledge of mental illnesses and their treatment, stigmatizing attitudes towards mental illnesses, or confidence in seeking help for self and offering help. | 4 |
| Information sources | 7 | Three authors (R.E., S.N., and A.A.) independently performed a literature search in two electronic databases: PubMed and PsycINFO. Two authors (R.E. and H.H) explored the Medline database for studies on MHL that had been published in any journal through July 2019 (without restriction to year of publication). A Boolean/phrase search was performed on each database, with search terms on the main concepts of interest: health literacy (concept 1), mental health (concept 2), and GCC countries (concept 3). | 4 |
| Search | 8 | Search strategy for pubmed: (Details of the search strategy are included in the Supplementary file 1).  (“Mental Health Literacy” OR “Mental health awareness” OR “Health knowledge” OR Knowledge OR “Mental Disorders Literacy” OR “Mood Disorders Literacy” OR “Depression Literacy” OR “Depressive Disorders Literacy” OR “Anxiety Literacy” OR “Bipolar Literacy” OR “Help seeking behaviour” OR “Help Seeking Behavior” OR “health seeking behaviour” OR “health seeking behavior” OR “seeking help” OR “help seeking” OR “Stigmatizing attitude” OR “Stigmatizing attitudes” OR stigma* attitude* OR “Attitude to health” OR percept* OR Believes) AND (“Disorder, Mental” OR “mental disorder*” OR “mental illness*” OR “[Mood](https://meshb.nlm.nih.gov/record/ui?ui=D019964) Disorder*” OR “Depressive Disorder*” OR depression OR Depressi* OR “anxiety disorder*” OR anxiety OR schizophrenia OR “obsessive compulsive disorder*” OR “Bipolar disorders” OR dementia OR “Alzheimer disease” OR Alzheimer) AND (“GCC countries” OR “Gulf council countries” OR “Arab states” OR “Middle East” OR Qatar OR Bahrain OR Oman OR Kuwait OR Saudi Arabia OR KSA OR “United Arab Emirates” OR UAE OR Arab)  Language English no limitations to date of publication | 4 |
| Study selection | 9 | Four authors R.E., S.N., A.A., and H.H. independently screened titles and abstracts, and excluded studies that were not relevant to the topic. They reviewed the full-texts of articles. First, database searches were exported into a master folder. All titles and abstracts were screened by R.E. and H.H. and then screened by S.N. or A.A. to assess eligibility for full-text printing and screening of references. Further, these authors independently screened all excluded titles and abstracts. If there was a disagreement, it was discussed with M.B. or N.E. to reach a final decision. | 5 |
| Data collection process | 10 | Independent data extraction of studies was performed by all four authors (R.E., S.N. A.A. and H.H.), to compare data and reach consensus. | 5 |
| Data items | 11 | The following were extracted from each one: country, title, authors, time of study, design, population group, sample size, outcome measures, and the Newcastle-Ottawa Scale (NOS) score of the study. | 5 |
| Risk of bias in individual studies | 12 | The quality of the studies and related bias were assessed by using NOS, adapted for cross-sectional studies [11]. This tool evaluated three quality parameters (selection, comparability, and outcome), divided across eight specific items. Each item on the scale was scored from one point, except for comparability, which can be adapted to the specific topic of interest to score up to two points. Thus, the maximum for each study is eight, with studies having less than four points identified as representing low quality. In order to minimize a subjective interpretation of bias in scoring the NOS, two independent authors should typically have scored each paper. All studies were assessed for quality in three domains: study selection, comparability, and outcome. | 5 |
| Summary measures | 13 | Studies must include at least one outcome measure, which was categorized as mental health attitudes (i.e., stigma, prejudice), knowledge of mental health (i.e., disorder and symptom recognition), or behavior regarding mental health (i.e., intended or actual help-seeking). | 6 |
| Synthesis of results | 14 | Results of each study were critically reviewed and the discussion was scrutinized for limitations and bias authors may have had highlighted in their studies. Following that we planned to combine and examine various related ideas in literature, to show how included results, outcomes, and limitations fit together, and present them in a unified form. | 6 |

Page 1 of 2

| **Section/topic** | **#** | **Checklist item** | **Reported on page #** |
| --- | --- | --- | --- |
| Risk of bias across studies | 15 | We used the items from CASP tool to draw limitations that were faced in this review. | 6 |
| Additional analyses | 16 | Not applicable, as all included studies were cross-sectional and consisted of high heterogenicity, hence this has prevented us from performing additional analysis in forms of meta-analysis | - |
| **RESULTS** | | |  |
| Study selection | 17 | We identified 341 studies in the initial search of databases. Next, we screened titles and ended with a total of 47. After removing 11 duplicates, we examined the abstracts of 36 potentially eligible studies, with nine of them excluded for not meeting the criteria for selection after fully reading the texts (three studies) or inaccessibility to the full text despite contacting the authors directly via email (six studies). Reference lists of these studies were screened as well, and finally 27 studies were included in this review. | 6 |
| Study characteristics | 18 | The included studies involved 16,391 participants who were primarily adults from the community. In six studies, participants were healthcare professionals [12,19,24,26,27,37], and five studies were conducted among college students [16,23,25,28,31,36]. In general, females constituted more than half of all study participants. Moreover, the majority of studies stated the definition of at least one component of MHL and explored the possible sociodemographic factors believed to influence the level of mental health knowledge, attitude, stigma, and MHL overall; specifically, gender, [22,31,35] marital status, level of education [21], ethnic groups [33].  Common demographic predictors of a lower level in recognizing mental illnesses across studies were younger age, unemployment, illiteracy, and female gender. To the contrary, one paper from Saudi Arabia showed that gender and type of education (medical and non-medical students) were not significantly associated with the level of MHL. However, such findings cannot be generalized, as they included students from only one university [23]. | 7-13 |
| Risk of bias within studies | 19 | The selection of adult participants in ‎reviewed studies was from different populations, but not usually justified. Most studies reported ‎specific inclusion and exclusion criteria. Probability sampling was conducted in few studies, ‎which renders results representative of the selected population, such as random sampling [38], ‎cluster probability [26,27], and multistage stratified sampling design [12,19][32-34]. The ‎remaining studies utilized nonprobability sampling from the community [13-15][22-25][28-31].‎  Studies were heterogeneous in terms of outcome measures. The vast majority of papers utilized valid and reliable measurement tools; these tools were piloted and tested within the populations, thereby making these studies less vulnerable to measurement bias. Some studies adapted validated tools such as community attitudes toward the mentally ill, the CAMI Scale [37,38], while others used measurement instruments that fulfilled the Diagnostic and Statistical Manual of Mental Disorders, Fifth Edition (DSMV) [26,27]. | 13-19 |
| Results of individual studies | 20 | Shown in table 1; which included the characteristics of the studies included in the systematic review | 8-13 |
| Synthesis of results | 21 | Using the detailed presentation of studies characteristics, we combined and examined ideas to develop an informed evaluation of the idea to explore the gap in mental health literacy across GCC nations by presenting several different viewpoints and/or ideas.; in order to form a coherent whole. | - |
| Risk of bias across studies | 22 | This review was limited to peer-reviewed articles published in English, which could result in bias. While extensive search was conducted, it is possible that relevant articles were not identified, as authors were using specific scientific databases. The evaluations may poorly assess the study quality, when details are not included in the reports. ‎ | 14-19 |
| Additional analysis | 23 | Quantifying the differences in means of levels of mental health literacy that were reported across included studies was not possible due to differences in measuring the outcomes and the scales used in addition to the selection of certain mental diseases to assess the level of mental health literacy related to them across different population groups. For example, adult females, health care providers or general adults. | 19 |
| **DISCUSSION** | | |  |
| Summary of evidence | 24 | Studies generally yielded average quality, based on the NOS assessment scores; this is likely due to potential sources of bias, which may compromise internal validity. three methodological issues could compromise the validity and reliability of the results. First, different terminologies were used, such as health literacy, knowledge, attitude, practice, health-seeking behavior, and stigma. Second, many included studies used different methods to assess recognition of mental illness, which can lead to measurement bias. Third, variations in the population under study including university students, adults, and healthcare providers. | 19-21 |
| Limitations | 25 | This review was limited to peer-reviewed articles published in English, which could result in bias. Countries in the GCC uses Arabic as the first language spoken, however, English is the second Language spoken and the formal language used in medical field including both practice as well as academic and scientific professional research. There, is only a few numbers database that covers articles written originally in Arabic language, but they mostly cover only limited numbers of articles from wide range of topic areas like art and engineering, but not exclusively for health nor mental health in specific. Hence choosing English was the best choice that would yield more studies in searching. While extensive search was conducted, it is possible that relevant articles were not identified, as authors were using specific scientific databases. The three databases selected were among the most commonly used in systematic reviews. And relating to similar literature on the topic from the region, these same databases were commonly and most frequently used. Never the less, including more data bases could have yield more articles, but it wasn’t feasible nor convenient to add more search engine in the allocated time of the review. Authors also did not include information from other sources, such as unpublished reports from educational institutions or relevant literature. Our authors did not contact other authors to clarify vague information in reviewed studies. Thus, the evaluations may poorly assess the study quality, when details are not included in the reports. | 22-23 |
| Conclusions | 26 | This review promotes common issues that shape and influence the level of MHL across GCC countries. These findings also suggest that there is a great need for interventions and public campaigns to both increase and promote MHL among the public. In addition, it emphasizes the need for robust cohort and interventional studies, given the importance of mental health, as well as its impact on the general well-being of the population. | 23 |
| **FUNDING** | | |  |
| Funding | 27 | The publication of this article was funded by the Qatar National Library. | 24 |

*From:*  Moher D, Liberati A, Tetzlaff J, Altman DG, The PRISMA Group (2009). Preferred Reporting Items for Systematic Reviews and Meta-Analyses: The PRISMA Statement. PLoS Med 6(7): e1000097. doi:10.1371/journal.pmed1000097

For more information, visit: **www.prisma-statement.org**.

Page 2 of 2
