# Supplementary material for: Mental health literacy in Arab states of the Gulf Cooperation Council: A systematic review
Source: PLoS One. 2021 Jan 7;16(1):e0245156. doi: 10.1371/journal.pone.0245156 (PMC7790272; doi:10.1371/journal.pone.0245156)
Supplement: Attachment — Submitted filename: Response to Reviewers.doc [file pone.0245156.s003.doc]

**Response to Reviewers**

**General Response to the Reviewers’ Comments:**

We are appreciative of the insightful comments raised by the Academic Editor and the reviewers. We believe we have responded to each of their comments. Where necessary, we have made revisions in the manuscript. These revisions are tracked. The line numbers referred to in our responses below refer to those in the tracked version of the manuscript.

**ACADEMIC EDITOR:**

Your manuscript would be much improved if you follow the PRISMA checklist for systematic reviews reporting. I suggest you in particular rewrite your abstract adding details of your studies inclusion and exclusion criteria.

**Response:** Thank you for your insightful observation, we have followed the PRISMA checklist and added more details in the abstract section. Please see lines 32-36.

**REVIEWERS COMMENTS**:
**Comment #1:**

Is the manuscript presented in an intelligible fashion and written in standard English?

Reviewer #1: No

Reviewer #2: Yes

**Response:**

Thank you for this feedback. We have engaged the services of a native English speaker to ‎review the entire manuscript and make the necessary corrections.‎

**Comment #2:** (**Reviewer #1)**

This is a relatively well designed, run and written systematic review focusing on Mental health literacy (MHL) in Arab states of the Gulf. Authors identified a moderate number of studies meeting their inclusion criteria showing that limited MHL was observed among participants, even within health care professionals, high level of stigma and negative attitude toward
mental health illnesses among the public. Negative beliefs and inappropriate practices were common, as well. The majority of studies yielded moderate to high risk of bias. It is appreciable authors explored a topic which has local but also some general interest. Provided language will be edited by some native English speaker (for example authors not reviewers perform screening) it might deserve publication.

What would need significant additional thoughts and work is the discussion paragraph, as it's often the case for systematic review and meta-analysis. Authors should try and expand the meaning of their findings over and above the mere technical results. Comparisons with available literature and alternative explanations attempts are currently quite poor.

**Response:**

Thank you for your insightful feedback. We have addressed your comments and the discussion section has been modified accordingly. Kindly see the details highlighted below:

**Lines 239-246 (discussion section)**

The discrepancies in terminology to define MHL, and the selection of different mental illnesses for included studies made it difficult to perform cross-country comparisons. This contradicted some findings from a review article, reporting on incomparable results with similar methodologies in countries of developed and developing regions: authors attributed the issues as being linked to religious beliefs, hindering interventions for populations with low MHL [39].

**Lines 279-294 (discussion section)**

Many studies suggest that a higher level of MHL is expected in the general population in developed countries, compared to their counterparts in developing regions. For instance, Furnham and Hamid in a systematic review (2014), examined a decade of research on MHL and found that citizens in developed countries had greater MHL: for example, the majority of reviewed studies showed that people had a greater recognition of depression than schizophrenia [8].

Authors justified their results in that the research from developing regions often used medical jargon and obscure language for the lay individual; in addition, restricted use of a written format for assessments rather than other forms of communications, such as audios and visuals, could complicate the situation [8]. Our study focused on the public, in which average MHL was found; these individuals were unable to recognize symptoms of common mental illnesses (e.g., depression and schizophrenia). A number of influencing factors could account for an opposing conclusion, such as literacy levels, cultural boundaries, and religious beliefs.

**Lines 306-312 (discussion section)**

Knowledge about mental health illness is lacking among the public as well as health care providers, in the available literature nowadays, despite the general perception that health care providers are more equipped to deal with patients suffering from mental illnesses. In this review we found low levels of MHL among physicians, nurses, and other related health care workers. These findings are related some studies that revealed limited knowledge and awareness of common mental disorders among health care providers, that coincides with false beliefs mostly acquired from their community and cultural environments [45,46].

**Lines 313-325 (discussion section)**

Internationally, mental health interventions were used in promotion models for individuals, schools, and communities [47]. A number of successful awareness campaigns that targeted the general public was reported in the UK, Norway, and the USA, in which media were used to reach larger audiences [48]. Additionally, an Australian trial set out to assess the effectiveness of an intervention called ‘First Aid,’ which showed remarkable results among adults in the workplace [49]. The use of such methods for improving MHL in developing countries requires further study. In this review, interventional studies were not identified in GCC countries, which can be explained by multilevel obstacles hindering strategy implementation, such as acceptability of interventions at the cultural level [50]. MHL levels in the GCC must be accelerated in the future.

**Lines 335-342 (conclusion section)**

This review promotes common issues that shape and influence the level of MHL across GCC countries. These findings also suggest that there is a great need for interventions and public campaigns to both increase and promote MHL among the public. In addition, it emphasizes the need for robust cohort and interventional studies, given the importance of mental health, as well as its impact on the general well-being of the population.

**Comment #3:** (**Reviewer #2)**

It is an important and topical issue. The review will help identify the gaps, however it will be helpful to identify some evidence informed interventions which could promote mental health literacy in specific population groups

**Response:**

Thank you for your insightful comment. We have addressed your comment in the discussion section. Kindly see the details highlighted below:

**Lines 313-325 (discussion section)**

Internationally, mental health interventions were used in promotion models for individuals, schools, and communities [47]. A number of successful awareness campaigns that targeted the general public was reported in the UK, Norway, and the USA, in which media were used to reach larger audiences [48]. Additionally, an Australian trial set out to assess the effectiveness of an intervention called ‘First Aid,’ which showed remarkable results among adults in the workplace [49]. The use of such methods for improving MHL in developing countries requires further study. In this review, interventional studies were not identified in GCC countries, which can be explained by multilevel obstacles hindering strategy implementation, such as acceptability of interventions at the cultural level [50]. MHL levels in the GCC must be accelerated in the future.

**Journal Requirements:**

**1-** When submitting your revision, we need you to address these additional requirements: Please ensure that your manuscript meets PLOS ONE's style requirements, including those for file naming.

**Response:** We have checked the style used in our manuscript against those required by PLOS ONE.

**2-** Please provide the full search string used, which at the moment is not included in the Supplementary file 1.

**Response:** We have provided the full search string used in the Supplementary file 1.
