# Supplementary material for: Mental health literacy in Arab states of the Gulf Cooperation Council: A systematic review
Source: PLoS One. 2021 Jan 7;16(1):e0245156. doi: 10.1371/journal.pone.0245156 (PMC7790272; doi:10.1371/journal.pone.0245156)
Supplement: Attachment — Submitted filename: Response to Reviewers.doc [file pone.0245156.s004.doc]

**Response to reviewers**

**General Response to Comments:**

We are appreciative of the raised comments and feedback by the Academic Editor and the reviewers, and made all possible efforts to respond to each of their comments. We have made minor revisions in the manuscript. These revisions are tracked and colored with red font. The line numbers mentioned in our responses below refer to those in the tracked version of the manuscript.

**ACADEMIC EDITOR:**

We suggest in particular you go through the PRISMA checklist providing explanations for all the items that were not met and include a synthesis in the limitations of the study. For example, you may wish to expand on the reasons why you only included articles in English and you did not consider publications in Arab Languages, as suggested by Reviewer 3. You may elaborate also on the choice to include a selected number of articles databases and how this may have restricted the number of publications considered in your review.

**Response:**

Thank you for your valued feedback, we have revised the PRISMA checklist and added more details providing explanations for all the items that were not met and included a synthesis in the limitations of the study.

Please look at the following items in the PRIMSA checklist:

14, 15, 16, 20, 21, 23, 25

Please look at their corresponding pages’ numbers as mentioned in the checklist as well

**REVIEWERS COMMENTS:**

From a study design standpoint, this is a relatively well conducted systematic review, but with some possible flaws. In my opinion, having decided to include only English language studies may have introduced a study selection bias as well as a publication bias. Why decide not to include also articles produced in the languages of the Arab member states of the GCC or other languages that can be spread in that geographical area?
I believe that a greater number of databases could have been explored (for example CINHAL and others of psychological and psychiatric interest such as PubPsych or PsyARTICLES and others). Furthermore, it may have had repercussions on the heterogeneity and transferability of the results. The possibility of introducing generic biases in the review was considered in the analysis of the results at line 265, but was not elaborated on in the discussion.
I believe it would have been more appropriate to use PRISMA in planning audit reporting and that this would also have been helpful in assessing the quality of the studies.
In general, the discussion of the results should be strengthened in relation to the results and potential limitations of the review itself. Authors should try and expand the meaning of their findings over and above the mere technical results.
The review is certainly useful in providing information, albeit not extensive, of the possible information gaps in the MHL area in GCC.

**Response:**

Thank you for your insightful feedback. We have addressed your comments and the manuscript has been modified accordingly. Kindly see the details highlighted below:

Lines 119-124:

Additionally, for the results synthesis in this systematic review, we have critically reviewed and scrutinized the results and discussion of each study for outcomes, limitations and bias that authors may have had highlighted in their studies. Following that we planned to combine and examine various related ideas in literature, to show how included results, outcomes, and limitations fit together, and present them in a unified form. Finally, we used the items from CASP tool to draw limitations that were faced in this review, and further elaborate on them in the discussion.

Lines 202-206:

Furthermore, Quantifying the differences in means of levels of mental health literacy that were reported across included studies was not possible due to differences in measuring the outcomes and the scales used in addition to the selection of certain mental diseases to assess the level of mental health literacy related to them across different population groups. For example, adult females, health care providers or general adults.

Lines 260-261:

in the Arab region, where mostly both men and women tend to link emotional and mental issues with religious and social believes.

Lines 268-271:

This might potentially create a real barrier in achieving accessible and effective mental health services to the public and especially to vulnerable groups within the GCC region who may struggle to reach out for their primary health provider voluntary.

Lines 281-297:

This review was limited to peer-reviewed articles published in English, which could result in bias. Countries in the GCC uses Arabic as the first language spoken, however, English is the second Language spoken and the formal language used in medical field including both practice as well as academic and scientific professional research. There, is only a few numbers database that covers articles written originally in Arabic language, but they mostly cover only limited numbers of articles from wide range of topic areas such as art and engineering, but not exclusively on health nor mental health in specific. Hence choosing English was the best choice that would yield more studies in searching. While extensive search was conducted, it is possible that relevant articles were not identified, as authors were using specific scientific databases. The three databases selected were among the most commonly used in systematic reviews. Relating to similar literature on the topic from the region, these same databases were commonly and most frequently used. Never the less, including more data bases could have yield more articles, but it wasn’t feasible nor convenient to add more search engine in the allocated time of the review. Authors also did not include information from other sources, such as unpublished reports from educational institutions or relevant literature. Our authors did not contact other authors to clarify vague information in reviewed studies. Thus, the evaluations may poorly assess the study quality, when details are not included in the reports

**JOURNAL REQUIREMENT:**

While revising your submission, please upload your figure files to the Preflight Analysis and Conversion Engine (PACE) digital diagnostic tool, <https://pacev2.apexcovantage.com/>. PACE helps ensure that figures meet PLOS requirements.

**Response:**

We have checked the style used in our manuscript against those required by PLOS

ONE using the recommended tools mentioned.
